# Widespread, perception-related information in the human brain scales with levels of consciousness

**DOI:** 10.1162/imag_a_00240

**Published:** 2024-07-29

**Authors:** Andrew D. Vigotsky, Rami Jabakhanji, Paulo Branco, Gian Domenico Iannetti, Marwan N. Baliki, A. Vania Apkarian

**Affiliations:** Department of Biomedical Engineering, Northwestern University, Evanston, IL, United States; Department of Statistics and Data Science, Northwestern University, Evanston, IL, United States; Department of Neuroscience, Northwestern University, Chicago, IL, United States; Center for Translational Pain Research, Northwestern University, Chicago, IL, United States; Department of Anesthesiology, Northwestern University, Chicago, IL, United States; Division of Biosciences, University College London, London, United Kingdom; Neuroscience and Behaviour Laboratory, Italian Institute of Technology, Rome, Italy; Shirley Ryan AbilityLab, Chicago, IL, United States; Department of Physical Medicine and Rehabilitation, Northwestern University, Chicago, IL, United States

**Keywords:** decoding, stimulus, perception, fMRI, propofol, consciousness

## Abstract

How does the human brain generate coherent, subjective perceptions—transforming yellow and oblong visual sensory information into the perception of an edible banana? This is a hard problem. According to the standard viewpoint, processing in groups of dedicated regions—identified as active “blobs” when using functional magnetic resonance imaging (fMRI)—gives rise to perception. Here, we reveal a new organizational concept by discovering that stimulus-specific information distributed throughout the whole brain. Using fMRI, we found stimulus-specific information across the neocortex, even in voxels previously considered “noise,” challenging traditional analytical approaches. Surprisingly, these stimulus-specific signals were also present in the subcortex and cerebellum and could be detected from across-subject variances. Finally, we observed that stimulus-specific signal in brain regions beyond the primary and secondary sensory cortices is influenced by sedation levels, suggesting a connection to perception rather than sensory encoding. We hypothesize that these widespread, stimulus-specific, and consciousness level-dependent signals may underlie coherent and subjective perceptions.

## 1 Introduction

fMRI has revolutionized how we study the brain by providing a noninvasive way to measure neural activity on a millimeter scale. This high spatial resolution allows for comparing blood oxygenation level-dependent (BOLD) activity within and between tasks or stimuli, helping uncover the functional properties of local neural circuits. Task-based fMRI studies typically use forward inference to identify task or stimulus-related brain areas by correlating each voxel’s activation time course with the temporal profile of the task. A contrast map is generated by differencing the brain activity of different tasks, which produces task or stimulus-specific localized blobs when thresholded. However, this analysis assumes that only the identified blobs contain reliable task-specific information (Hanson, 2022). In this report, we challenge this established assumption by revealing that stimulus-specific information is available throughout the neocortex, including regions previously identified as “noise” by univariate measures. This information can be uncovered by integrating signal over large swaths of voxels (~10,000 voxels). After discovering this pan-neocortical information content, we explored stimulus-specific information in the subcortex, cerebellum, and across-subject neocortical covariance. Additionally, we found that increasing levels of sedation degrade the omnipresent information. This brain-wide information reflects perceptual (conscious) processes and may play a role in identifying subjective, holistic perception from incoming sensory inputs, such as recognizing that an oblong yellow object is an edible banana.

Decoding can assess the statistical information contained in functional neuroimages, presumably arising from neural information, by transmuting brain activity into a single number that is, ideally, monotonically related to a stimulus of interest. This monotonicity facilitates the discrimination between the stimuli of interest and no interest, the performance of which indicates the amount of stimulus-specific information in the data. In this work, we leveraged these decoding ideas to uncover stimulus-specific information across the neocortex, subcortex, and cerebellum in studies with basic sensory stimuli. We demonstrate that such widespread sensory-specific information is sensitive to propofol-induced sedation.

## 2 Methods

### 2.1 Overview

**Fig. 1.**
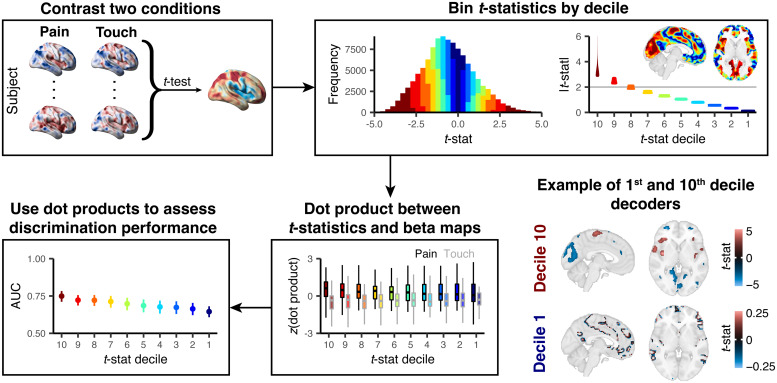
Generation of decoders to assess the presence of stimulus-related signals. Voxel-wise paired*t*-tests were performed on brain activity maps using a stimulus of interest (e.g., noxious stimulus eliciting pain) and no interest (e.g., touch). These*t*-statistics were then binned into deciles based on their absolute magnitudes. The gray line depicts the standard minimum threshold used to dichotomize brain regions that discriminate between stimuli (univariate “statistical significance” at α = 0.05, uncorrected for multiple comparisons). Each decile of*t*-statistics and their locations in the brain became a decoder. On the bottom right, we show examples of these decoders for the 10^th^(comprising large blobs) and 1^st^deciles (scattered voxels). Although the deciles were derived using the absolute value of*t*-statistics, the decoders incorporated the*t*-statistics’ signs. We then calculated dot products between the decoder derived from each decile and brain activity maps. These dot products are analogous to “linear predictors” from a regression model. Here, we*z*-scored the dot products within each decile for visualization purposes. We calculated AUCs based on these dot products, where higher dot products were assumed to correspond to the stimulus of interest. We used the 0.632 + bootstrap to obtain unbiased AUCs when testing our decoders.

Our paper is composed of two parts, for which we use six datasets with different sensory stimuli (Table S1;*N*= 293 subjects) (Baliki et al., 2009;Kandeepan et al., 2020;Liang et al., 2019;Naci et al., 2018;Pernet et al., 2015;Wager et al., 2013) to build simple decoding models using the*t*-statistics from standard general linear model (GLM) mass-univariate contrasts (Fig. 1, top left;Fig. S1).

The first part of our study examines decoding in 5 of these datasets, 4 of which contain 2 stimuli and 1 of which includes 4 stimuli, totaling 10 different stimulus pairs or contrasts$\left(4\left(\begin{array}{c} 2\\ 2 \end{array}\right)+\left(\begin{array}{c} 4\\ 2 \end{array}\right)=10\right)$. We binned the*t*-statistics by magnitude to create 10 decoders for each contrast; the first decoder comprised voxels with the highest*t*-statistics (10^th^decile) and the last decoder comprised voxels with the lowest*t*-statistics (1^st^decile) (Fig. 1, top right). We tested each decoder by calculating the dot product between the decoder (sign, magnitude, and location of*t*-statistics within a single decile; e.g.,Fig. 1, bottom right) and each brain activity map (general linear model (GLM)-derived maps of parameter estimates), yielding a weighted sum of stimulus-related activity across all voxels comprising the decoder (Fig. 1, bottom middle). We used the 0.623 + bootstrap to obtain unbiased out-of-sample estimates (like cross-validation) of between-subject areas under the receiver operating characteristic curve (AUC) to indicate discrimination performance (Fig. 1, bottom left), preventing our AUCs from being susceptible to overfitting. To succinctly describe our results, we meta-analyzed the resulting AUCs. We focused on comparisons between stimuli rather than comparisons to rest since the differences between stimuli are more subtle than the differences between each stimulus and rest; comparisons to rest can be found in the original studies.

The second part of our study uses the remaining dataset, in which participants listened to an auditory stimulus under different levels of propofol-induced sedation (Kandeepan et al., 2020;Naci et al., 2018). We performed similar analyses as those described above, and we compared AUCs across levels of sedation.

### 2.2 Datasets

The six datasets used in this paper are part of published studies and were either provided by their authors (Datasets 1–4) or downloaded from public repositories (Datasets 5 and 6). The studies that produced these datasets were conducted in accordance with the Declaration of Helsinki and were approved by local Institutional Review Boards. Datasets 1–4 consist of voxel-wise, whole-brain, stimulus-dependent GLM analysis activation maps. Datasets 5 and 6 consist of BOLD timeseries which were processed using standard fMRI methods described below. The preparation of these datasets has been previously described byJabakhanji et al. (2022).

#### 2.2.1 Dataset 1

Fifteen (15) right-handed adult subjects (mean age: 35 ± 11 years, 7 females). Subjects had no history of pain, psychiatric, or neurological disorders. fMRI data were collected while subjects received thermal stimuli across three temperatures: 47°C, 49°C, and 51°C. Subjects continuously rated, using a finger span device (Apkarian et al., 2001;Baliki et al., 2006), their pain from 0 (not painful) to 100 (worst imaginable pain) (“pain rating” stimulus). A control scan was performed while subjects used the finger span device to track a moving bar projected on the screen (“visual rating” stimulus; moving bar replicated for each subject the specific pain rating stimulus temporal pattern). The dataset includes one GLM beta map per subject per stimulus type. The dataset was previously described inBaliki et al. (2009).

#### 2.2.2 Dataset 2

Fifty-one (51) healthy right-handed adult subjects (age = 24 ± 2 years, 34 females). Subjects had no history of brain injuries, pain disorders, or psychiatric or neurological diseases. fMRI data were collected while subjects received painful heat stimuli on the right foot dorsum using an Nd:YAP laser, as well as tactile stimuli to the same area using electrical stimulation. Stimuli were not delivered at the same time. Perceived intensities were recorded for every stimulus and only the stimuli with matched perceived intensity for painful heat and touch were selected for GLM analysis. The dataset includes one activation map per subject per stimulus modality—painful heat and touch. The dataset was previously described inLiang et al. (2019)andSu et al. (2019).

#### 2.2.3 Dataset 3

Fourteen (14) healthy, right-handed, adult subjects (age = 20–36 years old, 6 females). fMRI data were collected while subjects received painful heat stimuli on the right foot dorsum using an Nd:YAP laser, tactile stimuli to the same area using electrical stimulation, visual stimuli using a white disk presented above the right foot, and auditory stimuli delivered via pneumatic earphones. Stimuli were not delivered at the same time. Perceived intensities were recorded for every stimulus and only the stimuli with matched perceived intensity across the four modalities were selected for GLM analysis. The dataset includes one activation map per subject per stimulus modality—painful heat, tactile, auditory, and visual. The dataset was previously described and published inLiang et al. (2019).

#### 2.2.4 Dataset 4

Thirty-three (33) healthy right-handed adult subjects (age = 28 ± 9 years, 22 females). Subjects had no history of pain, psychiatric, or neurological disorders. fMRI data were collected while subjects received thermal stimuli that varied in 1° Celsius increments across six temperatures from 44.3°C up to 49.3°C. Subjects then evaluated each stimulus as warm, and scored it from 0 (not perceived) up to 99 (about to become painful), or as painfully hot, and scored it from 100 (no pain) to 200 (worst imaginable pain). The dataset includes an average GLM activation map per subject per stimulus temperature, as well as the corresponding average stimulus ratings. When this dataset was applied dichotomously (pain vs. no pain), we averaged the brain activity maps from the painful and nonpainful conditions; we omitted subjects who had less than two brain activity maps for each condition, resulting in 29 subjects for dichotomous ratings. The dataset was previously described inWager et al. (2013)andWoo et al. (2015).

#### 2.2.5 Dataset 5

Two-hundred thirteen (213) healthy adult subjects (age = 24 ± 7 years, 101 females). Subjects had no history of physical or mental health conditions. fMRI data were collected while subjects performed a voice localizer task. Forty blocks of vocal sounds (20) and nonvocal sounds (20) interspersed with periods of silence were presented while the subjects lay silent and passively listened with their eyes closed in the scanner. This dataset was previously described byPernet et al. (2015). Raw fMRI data were downloaded from OpenNeuro (ds000158). We performed minimal preprocessing using the FMRIB 5.0.8 software library (FSL) (Jenkinson et al., 2012), MATLAB2018a, and in-house scripts. The following steps were performed: motion correction, intensity normalization, nuisance regression of six motion vectors, signal-averaged overall voxels of the eroded white matter and ventricle region, and global signal of the whole brain, and band-pass filtering (0.008–0.1 Hz) by applying a 4^th^-order Butterworth filter. All preprocessed fMRI data were registered to the 2 × 2 × 2 mm MNI152 template using a two-step procedure: the mean of preprocessed fMRI data was registered with a seven-degrees-of-freedom affine transformation (*x*,*y*,*z*,*α*,*β*,*γ*, and scale factor*k*) to its corresponding T_1_brain (FLIRT); next, transformation parameters were computed by nonlinearly registering individual T_1_brains to the MNI152 template (FNIRT). Combining the two transformations yielded a mapping from the preprocessed fMRI data to standard space. Task-related activation maps (vocal vs. silence, and nonvocal vs. silence) were derived from a whole-brain GLM regression analysis using the FMRIB Software Library (FSL) (Jenkinson et al., 2012;Smith et al., 2004;Woolrich et al., 2009).

#### 2.2.6 Dataset 6

Seventeen (17) healthy, adult subjects (4 women; age = 24 ± 5 years) took part in this study, which involved listening to a natural stimulus (5 minutes plot-driven audio story) and resting state (first 5 minutes of 8 minutes scan) while under different levels of anesthesia (Kandeepan et al., 2020;Naci et al., 2018). Sedation levels under propofol were determined by the Ramsey scale (awake, no propofol; light sedation, Ramsey = 3; deep sedation, Ramsey = 5; recover, Ramsey = 2, approximately 11 minutes after cessation of propofol) (Kandeepan et al., 2020). This dataset was previously described inKandeepan et al. (2020)andNaci et al. (2018). Raw fMRI data were downloaded from OpenNeuro (ds003171). We performed minimal preprocessing using the FMRIB 5.0.8 software library (FSL) (Jenkinson et al., 2012), MATLAB2018a, and in-house scripts. The following steps were performed: motion correction, intensity normalization, nuisance regression of six motion vectors, signal-averaged overall voxels of the eroded white matter and ventricle region, and global signal of the whole brain, and band-pass filtering (0.008–0.1 Hz) by applying a 4^th^-order Butterworth filter. All preprocessed fMRI data were registered to the 2 × 2 × 2 mm MNI152 template using a two-step procedure: the mean of preprocessed fMRI data was registered with a seven-degrees-of-freedom affine transformation (*x*,*y*,*z*,*α*,*β*,*γ*, and scale factor*k*) to its corresponding T_1_brain (FLIRT); next, transformation parameters were computed by nonlinearly registering individual T_1_brains to the MNI152 template (FNIRT). Combining the two transformations yielded a mapping from the preprocessed fMRI data to standard space. Task-related activation maps (auditory clip vs. resting state) were derived from a whole-brain GLM regression analysis using R.

### 2.3 Decoder construction and evaluation

Brain activity maps were masked to include only neocortical gray matter voxels using the Harvard-Oxford neocortical mask thresholded at 0.5. For each contrast, we performed a voxel-wise paired*t*-test using two brain activity maps from each subject, resulting in a*t*-statistic for each voxel in the gray matter. The*t*-statistic map was then binned into deciles by |*t*|—decile 10 contained the highest absolute value*t*-statistics (the “most significant”) and decile 1 contained the lowest absolute value*t*-statistics (the “least significant”). These unthresholded, deciled*t*-statistic maps served as our decoders.

We evaluated the decoders ($\text{D}\in {\mathbb{R}}^{p\times 10}$) by multiplying them with the brain activity maps of interest (${\text{B}}_{\text{I}}\in {\mathbb{R}}^{n\times p}$) and no interest (${\text{B}}_{\text{NI}}\in {\mathbb{R}}^{n\times p}$), for*p*voxels and*n*subjects. This resulted in two matrices of dot products between the decoders and brain activity maps: one matrix of dot products from the activity maps of interest (${\text{R}}_{\text{I}}={\text{B}}_{\text{I}}\text{D}$) and one matrix of dot products from the activity maps of no interest (${\text{R}}_{\text{NI}}={\text{B}}_{\text{NI}}\text{D}$). The columns of${\text{R}}_{\text{I}}$and${\text{R}}_{\text{NI}}$were then compared to calculate an AUC via the Mann–Whitney U-statistic ($AUC=U_{1}/n^{2}$). That is, column 1 in${\text{R}}_{\text{I}}$was compared with column 1 in${\text{R}}_{\text{NI}}$, column 2 in${\text{R}}_{\text{I}}$was compared with column 2 in${\text{R}}_{\text{NI}}$, and so on for all 10 columns, producing 10 AUCs—one for each decile. In doing so, we treated the subjects as dependent for decoder training (paired*t*-test) but independent for testing.

We constructed and tested all decoders using the 0.632 + bootstrap method with 100 replicates, which provides unbiased estimates of out-of-sample performance (Efron & Tibshirani, 1997). Briefly, the 0.632 + bootstrap was performed as follows:

1. Train and test a model using the original sample. Let the resulting AUC be called the “apparent” AUC,${\hat{\theta }}^{app}$.
2. Generate*b*bootstrap samples by resampling the original sample with replacement. Note, each bootstrap sample contains approximately1−1e=63.2%of the original sample. For each of the*b*bootstrap samples, we train the model on the bootstrap sample and test the model on the ~36.8% of individuals not part of the bootstrap sample. Let this AUC estimate be the “leave-one-out” (out-of-sample) bootstrap AUC,${\hat{\theta }}_{i}^{boot}$.
3. Average the resulting out-of-sample bootstraps,${\hat{\theta }}_{•}^{boot}=\frac{1}{b}\sum_{i=1}^{b}{\hat{\theta }}_{i}^{boot}$.
4. Obtain the 0.632 + estimate.
  - Calculate the relative overfitting rate,
$$\hat{R}=\left\{ \begin{array}{cc} & 1,{\hat{\theta }}_{\cdot }^{boot}\leq 0.5\\ ({\hat{\theta }}^{app}-{\hat{\theta }}_{\cdot }^{boot})/({\hat{\theta }}^{app}-0.5), & {\hat{\theta }}^{app}>{\hat{\theta }}_{\cdot }^{boot},\\ & 0,\;\;otherwise \end{array}\right.$$
    where 0.5 indicates no information in the decoder.
  - Calculate the weight to adjust the 0.632 estimate,
$$\hat{w}=\frac{0.632}{1-0.368\hat{R}}.$$
  - Calculate the 0.632 + estimate,
$${\hat{\theta }}^{0.632+}=\left(1-\hat{w}\right)\cdot {\hat{\theta }}^{app}+\hat{w}\cdot {\hat{\theta }}_{•}^{boot}.$$

Note that we did not adjust${\hat{\theta }}_{•}^{boot}$with$\text{max}\left({\hat{\theta }}_{•}^{boot},0.5\right)$in 4c as commonly done (Efron & Tibshirani, 1997;Noma et al., 2021), since this would create a floor effect such that${\hat{\theta }}^{0.632+}\geq 0.5$, which would downwardly bias our variance estimates in the next step. This results in${\hat{\theta }}^{0.632+}$estimates that are identical to estimates with the adjustment when${\hat{\theta }}^{0.632+}\;>\;0.5$, but allows our estimates to dip below chance (AUC = 0.5) since it removes the floor effect.

Variances and covariances of the AUCs were estimated using a nested bootstrap with 500 replicates in the outer loop and 100 replicates in the inner loop (Noma et al., 2021), totaling 500 × 100 = 50,000 replicates in each study. All inner and outer bootstraps were performed on the subject level. This sampling was carried out on Northwestern University’s High Performance Computing clusters (Quest), and took ~12 hours to complete using 50 cores.

### 2.4 Meta-analysis

We performed a single-paper meta-analysis to consolidate our results (McShane & Böckenholt, 2017). First, all AUCs were “squeezed” or shrunken toward 0.5 to avoid boundary effects (Smithson & Verkuilen, 2006),

$${\hat{\theta }}^{*}=\frac{\left(n-1\right)\cdot {\hat{\theta }}^{0.632+}\;+0.5}{n},$$

where*n*was the total number of brains used (i.e., twice the number of participants). Next, the 0.632 + bootstrap-estimated AUCs and their bootstrapped replicates were logit transformed, and the logit-transformed bootstrap replicates were used to generate a 100 × 100 variance–covariance matrix of sampling errors. The logit-transformed AUCs were used as the response variable in a multivariate, multilevel linear meta-regression (metafor, version 3.8-1) (Viechtbauer, 2010). This allowed for properly accounting for within-study dependence, including the dependence between deciles in a single contrast (e.g., decile 1 and decile 2 in Study 1) and the dependence between contrasts in Study 3 (e.g., decile 1 in touch vs. pain and decile 2 in visuomotor vs. pain). We were principally interested in the effect of decile on discrimination performance; we treated decile continuously and used it as a linear moderator (fixed effect). Similarly, decile was treated continuously in the random-effect specification, wherein contrasts were nested within studies. Finally, we performed a meta-analysis that combined the neocortex, subcortex, and cerebellum findings from contrasts 1–9, for which we used 270 × 270 variance–covariance matrices.

### 2.5 Across-subject decoding with principal components analysis (PCA)

To analyze the task relevance of the across-subject variance–covariance structure, we performed PCA on the pain beta maps from Study 2 using singular value decomposition on a column-wise*z*-scored${\text{B}}_{\text{I}}$. To limit the number of principal components (PCs), we performed parallel analysis by generating surrogate data—100 null datasets—and calculating the variance explained by each null component, against which we compared our observed explained variance. Within each surrogate dataset, we performed discrete Fourier transforms on each beta map, scrambled their phases, and performed the inverse Fourier transform (Lancaster et al., 2018). This enabled us to maintain identical spatial frequency content and similar autocorrelation functions to the original beta maps—this was important since autocorrelation easily gives rise to spurious correlations (Ernst et al., 2017), which can affect dimensionality estimates (Cordes & Nandy, 2006). We used the number of real PCs that fell above the noise floor as determined by the parallel analysis.

After establishing with the whole sample that four PCs fell above the noise floor, we used the bootstrap 0.632 + to fit PC-based decoders. In each training set, we column-wise*z*-scored${\text{B}}_{\text{I}}^{\text{train}}$and performed PCA to obtain the top four PCs. We then independently*z*-scored the brain activity maps in the test set,${\text{B}}_{\text{I}}^{\text{test}}$and${\text{B}}_{\text{NI}}^{\text{test}}$, projected them into the four-dimensional PC space of${\text{B}}_{\text{I}}^{\text{train}}$, and calculated their respective Kullback–Liebler divergence from${\text{B}}_{\text{I}}^{\text{train}}$:

$$\begin{array}{l} {\text{D}}_{\text{KL}}\left({\text{B}}^{\text{test}}||{\text{B}}_{\text{I}}^{\text{train}}\right)=\frac{1}{2}(\text{tr}\left(\Sigma_{\text{train}}^{-1}\Sigma_{\text{test}}\right)-4+{\left(\mu_{\text{train}}-\mu_{\text{test}}\right)}^{T}\frac{}{}\\ \;\;\;\;\;\;\;\;\;\;\;\;\;\;\;\;\;\;\;\;\;\;\;\;\;\;\;\;\;\;\;\;\;\;\;\;\;\;\;\;\;\;\;\;\;\;\;\Sigma_{\text{train}}^{-1}\left(\mu_{\text{train}}-\mu_{\text{test}}\right)+\ln\left(\frac{\det\Sigma_{\text{train}}}{\det\Sigma_{\text{test}}}\right)), \end{array}$$

whereμandΣwere calculated in the PC space. A geometric depiction of this operation is shown inFigure 3. If${\text{B}}_{\text{I}}^{\text{test}}$had a lower KL than${\text{B}}_{\text{NI}}^{\text{test}}$, that test sample received a 1; otherwise, the test sample received a 0. The final bootstrap 0.632 + estimate thus represents the expected out-of-sample classification accuracy.

As a point of comparison, we also used*t*-statistic decoders based on the entire neocortex (i.e., they were not binned by decile). For these decoders, the Kullback–Liebler divergence was assessed using the univariate response distributions. This ensured that the*t*-statistic decoder was assessed using the entire sample, akin to the PCA decoder. The correlation between the PCA and*t*-statistic decoding accuracies was estimated using the accuracies’ bootstrap replicates.

### 2.6 Perturbations

#### 2.6.1 Noise

Since voxels with low signal-to-noise ratios (i.e., low*t*-statistics) were capable of decoding, we aimed to evaluate this finding’s boundary conditions. Each brain activity map contains a correlation coefficient*r_i_*for each voxel*i*, along with a*t*-statistic*t_i_*. We started with a brain of*t*-statistics, to which we added Gaussian noise ($N\left(0,c|t_{i}\text{​}|\right)$, where*c*∈ {0,1,2,3,4,5}). This procedure ensured that the noise added to each voxel was proportional to its signal-to-noise ratio to avoid biasing the regions with high signal. The*t*-statistics with added noise were then converted to Pearson’s*r*, on which we performed the decoding. Noise was added within each 0.632 + bootstrap replicate such that the resulting AUCs were averaged over 500 iterations (as opposed to 100 for other analyses) of added noise.

#### 2.6.2 Voxel sampling

Since the ability to decode with low signal-to-noise voxels likely arises from integrating over several small bits of information, we randomly subsampled voxels. The full decoders contained approximately 10,735 voxels per decile, which could come from anywhere within our gray matter mask. We built and assessed decoders by subsampling the brain activity maps, such that the resulting decile-based decoders contained 100, 250, 500, 1,000, 2,500, 5,000, 7,500, and 10,735 voxels each. Voxels were sampled within each 0.632 + bootstrap replicate such that the resulting AUCs were averaged over 500 iterations (as opposed to 100 for other analyses) of sampled voxels.

### 2.7 Anatomical specificity

#### 2.7.1 Neocortex, subcortex, and cerebellum

Neocortical, subcortical (thalamus, striatum, hippocampus, and amygdala), and cerebellar gray matter voxels were extracted from each brain activity map. The neocortical gray matter mask contained 112,651 voxels, the subcortical mask contained 6,882 voxels, and the cerebellar cortex mask contained 17,142 voxels. Since decoding power is sensitive to the number of voxels, we randomly subsampled 6,882 voxels (or fewer for studies that were further masked) from each mask to control for number of voxels. This subsampling was completed within each 0.632 + inner bootstrap replicate.

#### 2.7.2 Neocortical gray matter, white matter, and cerebral spinal fluid

Neocortical gray matter (GM), white matter (WM), and cerebral spinal fluid (CSF) voxels were masked using the Harvard–Oxford atlas with conservative thresholds: 112,651 for GM, 61,324 for WM, and 1,926 for CSF. Within each study, we controlled for the number of voxels by resampling 1,925 voxels (since 1,926 < 61,324 < 112,651) from GM and WM within each bootstrap run.

### 2.8 Anesthesia decoders

The anesthesia dataset employed a naturalistic audio stimulus and thus does not have a task vector. Moreover, this was the only task performed. As such, we compared each anesthesia level’s task (naturalistic listening) to rest. We used the average auditory cortex activity from the training sample as the task vector to facilitate this. This analysis is obviously circular within the training sample. However, the testing is not circular because the training sample’s brain activity was used as the vector in the testing sample and decoding was assessed based on the resulting brain activity maps. To extract the auditory cortex vector, we defined a region of interest (ROI) based on the Neurosynth association map for “auditory,” which was thresholded using a*z*-score of 12.

#### 2.8.1 Decile decoders

Decile-based decoders for the anesthesia dataset were created similarly to the other datasets. To summarize the performance within each decile, we fit a single generalized least squares model on the logit-transformed AUCs from all anesthesia states,**y**. To do so, all AUCs were “squeezed” toward 0.5 like they were for the meta-analysis. Our weight matrix,**W**, was defined as the inverse of the variance–covariance matrix of the logit-transformed bootstrapped AUCs,**$\hat{\Sigma }$**. The parameter estimates,$\hat{\beta }$, and their standard errors were calculated as

$$\hat{\beta }={\left({\text{X}}^{\text{T}}\text{WX}\right)}^{-1}{\text{X}}^{\text{T}}\text{Wy}$$

$$\begin{array}{c} \text{SE}\left[\hat{\beta }\right]=\sqrt{\text{diag}\left({\left({\text{X}}^{\text{T}}\text{WX}\right)}^{-1}{\text{X}}^{\text{T}}\text{W}\hat{\Sigma }\text{WX}{\left({\text{X}}^{\text{T}}\text{WX}\right)}^{-1}\right)}\\ =\sqrt{\text{diag}\left({\left({\text{X}}^{\text{T}}\text{WX}\right)}^{-1}\right)}, \end{array}$$

where**X**is the design matrix,

$$\text{X}=\left[\begin{array}{cc} 1 & x^{\text{decile}} \end{array}\right]⊗\left[\begin{array}{cccc} x^{\text{awake}} & x^{\text{light}} & x^{\text{deep}} & x^{\text{recover}} \end{array}\right].$$

This differs from standard weighted least squares (a diagonal weight matrix) but has more favorable properties since it accounts for covariation.

#### 2.8.2 Region of interest decoders

We created posterior cortex, anterior cortex, visual cortex, and motor cortex ROIs using the Harvard–Oxford neocortical atlas, thresholded at 25%. The posterior cortex was specified as areas 20–22; the anterior cortex, areas 1, 4, and 5; the visual cortex, areas 36, 40, and 48; and the motor cortex, area 7. In addition, we used the same auditory cortex ROI as described above. Again, the averaged auditory ROI time course from the training sample was used as the task vector. A decoder was created using the*t*-statistics from each ROI (without deciles), which was fit and tested using the same approach as the decile decoders.

#### 2.8.3 Functional connectivity

Pearson correlation coefficients were used to calculate within- and intersubject (ISFC) functional connectivity between the auditory cortex and the other ROIs, during both the auditory task and rest within each level of anesthesia. Within-subject functional connectivity was calculated by averaging the time course between all voxels within each ROI, calculating the correlation between the auditory ROI and all other ROIs, converting from Pearson’s*r*to Fisher’s*z*, averaging across participants, and then converting back to Pearson’s*r*. ISFC was calculated similarly to previous work (Simony et al., 2016). When calculating subject 1’s ISFC, we correlated subject 1’s auditory cortex time course with the average time course of, for example, posterior cortex from subjects 2–*n*. This was repeated for all subjects. The resulting auditory cortex–posterior cortex ISFCs were averaged using Fisher’s*z*to obtain the final estimate of the auditory cortex–posterior cortex ISFC. Again, this was repeated for the anterior, visual, and motor cortex; ISFC was also measured between auditory cortices across all subjects.

#### 2.8.4 Statistical inference

Rather than relying on null hypothesis significance testing (NHST) and the thresholds intrinsic to it (Amrhein et al., 2019;McShane et al., 2019), our statistical inferences rely on effect estimation by quantifying the effect (e.g., AUC) and its uncertainty given our statistical models’ assumptions.

## 3 Results

### 3.1 Stimulus-specific information is widespread across the human neocortex

**Fig. 2.**
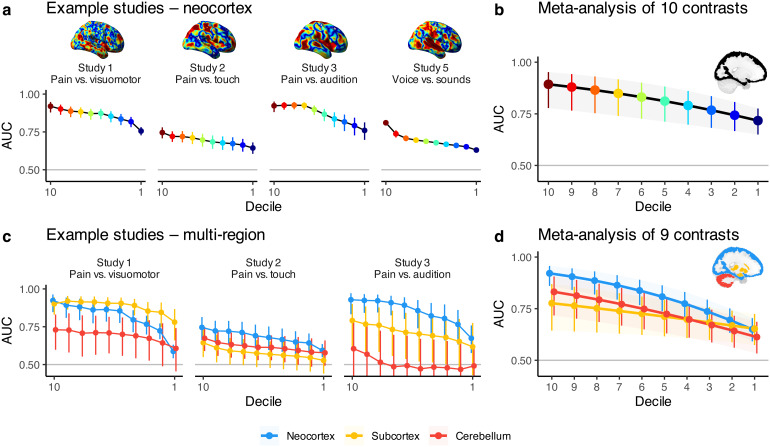
Stimulus-specific information across the neocortex, subcortex, and cerebellum. (a, b) We first analyzed stimulus-relevant information using the entire neocortex. (a) Four example studies of neocortical decoding performance across all*t*-statistic deciles. Although all 4 studies have stimulus-specific information in all 10 deciles, the degree to which the stimuli can be discriminated differs slightly between studies. Error bars indicate ± SE. (b) A mixed-effects meta-analysis across 10 contrasts reveals that all 10 deciles can, on average, discriminate between stimuli. The ability to discriminate between stimuli implies the presence of task-specific information, meaning that even voxels with*t*-statistics close to 0 (decile 1) contain marked task-specific information. Error bars indicate ± CI_95%_. (c, d) After finding task-specific information across the neocortex, we probed for task-specific information in the subcortex and cerebellum. To facilitate fair comparisons between structures, we matched the number of voxels using random subsamples (seeMethods), resulting in slightly different neocortical results. (c) Three example studies demonstrate marked differences in regional task-specific information. In Study 1, the relative task-specific information in the neocortex shifts from being closer to the subcortex to the cerebellum. In Study 2, all three regions are more similar, while in Study 3, there is a consistent pattern with the three areas being starkly different. (d) After controlling for the number of voxels in the neocortex, subcortex, and cerebellum, a meta-analysis across nine contrasts (excluding Study 5) reveals that task-related information exists across all regions and deciles. Task-related information in the neocortex dominates for higher deciles, but this superiority vanishes in smaller deciles (see intercept and slope contrasts inTable S5). Note that the difference in error bar widths between the three conditions partly reflects the nonlinearity of the AUC scale, which will tend to have more precise estimates near the floor (0) and ceiling (1). Error bars indicate ± CI_95%_.

Decoding performance was consistently above chance (AUC > 0.5) for all deciles across 9 out of 10 contrasts. Despite univariate*t*-statistics in the lowest decile being close to 0, the lowest decile’s decoding performance was only marginally poorer than the highest decile’s decoding performance (Fig. 2a, b;Table S2). Therefore, regions in neocortical gray matter commonly considered not to be involved in stimulus processing (i.e., orthogonal to the stimulus in univariate analyses) contain instead robust stimulus-related information. These findings complement recent work using statistical learning to optimize voxel weights for predictive performance (Cox & Rogers, 2021;Kumar et al., 2020;Mohr et al., 2015;Rish & Cecchi, 2017;Rish et al., 2012) and demonstrate that the presence of information is far more distributed across the brain than previously thought. Moreover, our results show for the first time how accessible this information truly is: our models use plain mass-univariate*t*-statistics, without any regularization or consideration of their joint distribution. Regularization and multivariable modeling are unnecessary, and even voxels with*t*-statistics close to 0 can jointly discriminate stimuli from one another quite well (meta-analytic AUC > 0.7). Therefore, our results indicate the presence of rich stimulus-related information throughout the neocortex, which degrades slowly as a function of the univariate signal-to-noise metric (*t*-statistic deciles).

### 3.2 Potential confounds that may account for the widespread stimulus-related information

When decoding stimuli from neuroimages, one cannot infer the causal role of the predictors (voxels) in the decoding outcome (task) (Jabakhanji et al., 2022;Vigotsky et al., 2024). There are many reasons why information may be present in acausal structures. For example, physiological and nonphysiological noise may have task specificity (Liu, 2016). We attempted to rule out such confounds by decoding stimuli using white matter and cerebrospinal fluid (cf. gray matter). Decoding performance was generally poor in these regions (Fig. S2), suggesting that these control structures contain less signal than the neocortex. In addition, white matter and cerebrospinal fluid decoding performance substantially covaried (*r*= 0.7), but they only weakly covaried with gray matter, implying vastly different signal sources between these structures (Fig. S2). Finally, head motion artifact—another potential candidate of task-related noise that contaminates the BOLD signal—only minimally correlated with decoder responses; orthogonalizing the decoders’ dot products to head motion only slightly decreased discrimination (maximum ΔAUC < 0.05). Thus, our observed effects are unlikely attributable to task-specific, non-neural confounds.

### 3.3 Decoder performance’s dependence on added noise

To assess the general sensitivity of the decoders, we built them using different numbers of voxels and different amounts of added noise. Decoders with fewer voxels performed poorly and were more sensitive to added noise (Fig. S3). As one might expect, the ability to successfully decode using voxels with low*t*-statistics is principally attributable to the number of included voxels. There is a continuum of explanations for why this might be the case. On one extreme, each voxel may contain a tiny amount of task-specific information. Integrating many small bits of information allows one to accumulate enough information to discriminate between stimuli. On the other extreme, since*t*-statistics are empirically derived and thus will not be stable across samples, “signal” voxels may mix with “noise” voxels, creating instability in our deciles. In this case, by sampling more voxels, we are more likely to capture “signal” voxels within the “noise” decile(s) to enable successful decoding.

### 3.4 Stimulus-specific information is widespread across the subcortex and cerebellum

Next, we tested whether the*t*-statistic information content is specific to the neocortex. Our analyses revealed that information is present throughout both the subcortex and the cerebellum, even where*t*-statistics are approximately 0 (Figs. S4 and S5;Tables S3 and S4). Cerebellar information varied more between task pairs than the neocortex (three examples shown inFig. 2c;Tables S2 and S4). These results complement recent work byNakai and Nishimoto (2022), who used the subcortex and cerebellum to decode 103 cognitive tasks using a within-subject approach based on more complex models trained using statistical learning. In contrast, we used*t*-statistics from regional activity maps to decode across rather than within subjects. Our meta-analysis across task contrasts showed that the performance of the subcortical and cerebellar decoders was only slightly inferior to that of the neocortex-based decoders, even after controlling for the number of voxels (Fig. 2d;Fig. S6;Table S5). Overall, we observed that subcortical and cerebellar structures contain widespread, task-specific information, demonstrating that information spread is not restricted to the neocortex but is present across the entire human brain.

### 3.5 Task-specific information is contained within the across-subject variance structure

**Fig. 3.**
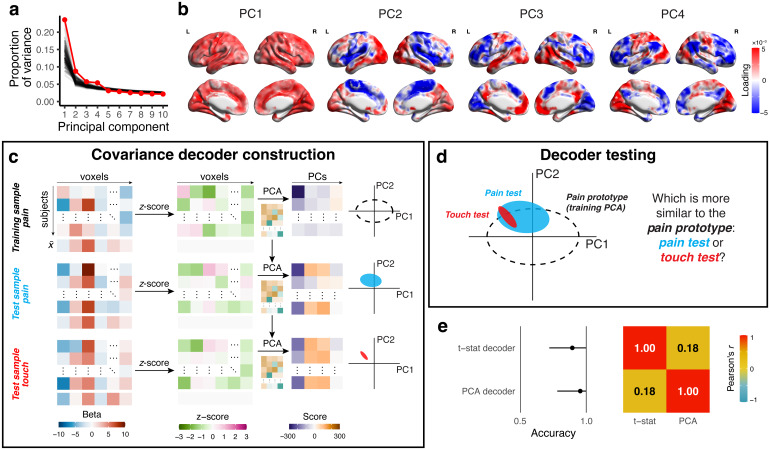
Principal components analysis reveals stimulus-related covariance across participants that is orthogonal to the*t*-statistic decoding. (a) We performed principal components analysis (PCA) across subjects using the brain activity maps for the pain condition of Study 2 (Liang et al., 2019). Parallel analysis indicated that the first four principal components (PCs) were above the noise floor. (b) Each PC has a unique corresponding spatial structure; their covariance structure is neither strictly local nor contiguous. (c) We used the PCs from the pain condition as a decoder (a “prototype”) by*z*-scoring the brain activity maps from out-of-sample pain and touch conditions. Next, we projected them onto the four PCs from the training sample.$\bar{x}$indicates the column mean, which is transformed to 0 when the brain activity maps are*z*-scored; that is, all data that go into the PCA are centered and thus have a mean of 0. (d) We compared the out-of-sample PC score distributions with the pain prototype distribution using Kullback–Liebler divergence. Whichever test sample had the lower divergence “won,” and the relative frequency of wins in the pain condition was the accuracy. (e) On average, the PCA decoder outperformed the*t*-statistic decoder (accuracy = 0.95 vs. 0.89). Importantly, the accuracies were only weakly correlated (*r*= 0.18).

Our*t*-statistic analysis relies on the stimulus’s so-called main effect within each voxel. However, stimulus-relevant information may exist beyond the main effect—namely, in the variance structure. We evaluated the presence of higher-order, stimulus-specific information by performing principal components analysis (PCA) on data from one of the larger studies with equisalient stimuli (Study 2, pain vs. touch;*n*= 51) (Liang et al., 2019). We extracted four principal components (PCs) (Fig. 3a, b), with which we decoded across-subject variance. We fit PCs in the training sample to assess the across-subject variance–covariance structure of the pain condition. Next, we projected the test sample’s pain and touch activation maps into the same PC space (Fig. 3c).

By comparing the multidimensional structure of the test samples with the original training sample, we could distinguish pain activation maps from touch activation maps with 95% accuracy. Interestingly, this suggests that unique patterns of stimulus-related activity (PCs) differ across participants. This perspective is partly supported by visual inspection of the PCs, which appear physiological rather than reflecting motion or registration artifacts, where one might expect edge effects (Fig. 2b). From a constructivist viewpoint, one interpretation is that there is a many-to-one relationship between brain activity and gross percepts, consistent with the notion of*degeneracy*(Barrett, 2017;Edelman & Tononi, 2000). In our view, a related idea from motor control,*abundance*, complements and expands on degeneracy by providing a teleological explanation: since a one-to-one relationship between neural activity and percepts would be overly prescriptive and, as a result, inflexible and unstable, a many-to-one relationship allows for the nervous system to organize and tune itself to robustly complete a task (Latash, 2000)—in this case, generating a percept. Many different combinations of brain activity patterns may be sufficient to create the perception of a banana. Since these discordant patterns are generalizable, exploring individual differences may allow for the discovering of neurocognitive rules. This analysis is only weakly correlated with the*t-*statistic decoder’s result (Fig. 3d) and demonstrates the presence of ample stimulus-specific information in the higher (statistical) moments of brain activity maps—that is, the variance and covariance structure.

Understanding the nature of this brain-wide information is more challenging than identifying its existence. Recent work in mice demonstrates widespread cortical dynamics as necessary for behavior—preventing local activation clusters impairs performance, suggesting a functional rather than epiphenomenal role (Pinto et al., 2019). If activation across the entire brain is necessary for task performance, it is more likely that the information we detected across brain regions is complementary rather than redundant. In other words, different brain regions capture distinct properties of the task or stimulus. However, the question arises as to whether this widespread information is involved in conscious perception or is simply a correlate. To address this, we will now link these findings to the question of how conscious percepts arise from neural activity.

### 3.6 Widespread, stimulus-specific information in the neocortex scales with consciousness

Neurophysiological theories of consciousness posit that brain-wide information sharing is necessary but not sufficient for consciousness (Tononi & Koch, 2015). Conceivably, the association between information sharing and consciousness (Casali et al., 2013) suggests that stimulus-specific brain-wide information should attenuate with increasing levels of sedation. Information cannot be omnipresent if it is not readily shared across the brain. But how do states of consciousness interact with stimulus-specific, brain-wide information content? To assess this, we analyzed a dataset in which individuals listened to an auditory stimulus (5-minute audio from a movie) under different levels of propofol-induced sedation (Kandeepan et al., 2020;Naci et al., 2018). Since there was no auditory stimulus vector, we averaged participants’ auditory cortex time courses to serve as the stimulus vector. We used a separate resting-state scan as a negative control.

**Fig. 4.**
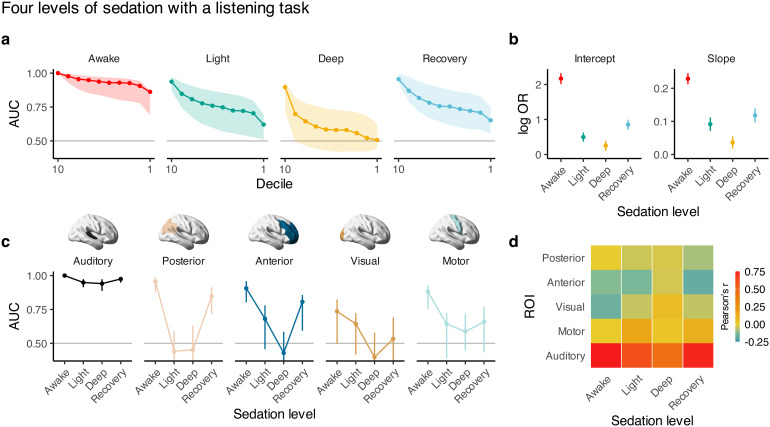
Sedation affects the neocortical distribution of stimulus-relevant information in a region-specific manner. (a) Widespread cortical stimulus-relevant information decreases with deeper levels of sedation, as indicated by the decrease in AUCs from awake to light to deep and the increase in AUC from deep to recovery. (b) Intercepts (defined by decile = 1) and slopes of the curves in (a) reveal stark decoding differences between levels of anesthesia. Awake’s first decile (intercept) has the strongest performance of the different levels of sedation. Its slope (on the logit scale; OR = odds ratio) is also greatest since it is more difficult to improve performance as AUC → 1. (c) The primary and secondary auditory cortices retain information across sedation levels. In contrast, auditory-related information in other cortical regions drops with increasing sedation levels. (d) Stimulus-specific intersubject functional connectivity decreases with deeper sedation; these effects were highly incompatible with the null hypothesis of no change across sedation levels ($\chi^{2}\left(15\right)=115,P_{\text{perm}}<0.001$). All error bars indicate ± CI_95%_.

Consistent with our analyses above,*t*-statistic decoding showed that stimulus-related information was omnipresent across the neocortex when participants were awake. However, this information degraded with increasing levels of sedation and was partially restored while recovering from sedation (Fig. 4a, b). We performed a region-of-interest (ROI)-based analysis to complement the region-agnostic decile analysis. In the awake state, different regions exhibited distinct abilities to discriminate the stimulus from resting state, with the auditory cortex exhibiting the greatest discrimination. Moreover, the auditory cortex’s stimulus-specific signal was invariant to sedation level, but stimulus-specific information degraded with deeper levels of sedation across all other ROIs (posterior, anterior, visual, and motor cortices) (Fig. 4c). Our findings imply that brain-wide, stimulus-specific information content is related to subjective perception rather than the simple encoding of the stimulus features.

Our results are consistent with the recent report byTauber et al. (2024), who used Utah arrays to record from macaques’ auditory, associative, frontal cortices during an auditory stimulus. They found that auditory cortex activity was intact following propofol administration, but associative and frontal cortex firing was drastically weakened. These findings are also consistent with fMRI in macaques (Uhrig et al., 2016). Whether using electrodes or fMRI, it appears that local auditory sensory processing remains intact with increasing levels of sedation while activity elsewhere degrades.

### 3.7 Intersubject functional connectivity scales with consciousness

The brain’s modularity and interconnected functional and structural networks must underly our observed brain-wide distribution of information (Eguiluz et al., 2005;Knosche & Tittgemeyer, 2011;Sporns et al., 2000a,2000b). In particular, long-range connections and the consequently arising small-world networks provide a mechanism for efficient information sharing. The properties of these networks are thus likely to be critical for how information is communicated and captured across the brain. We elucidated the relevance of functional connectivity to our findings by examining intersubject functional connectivity (ISFC) using the auditory cortex as a seed. We quantified the temporal relationship between a participant’s auditory cortex and all other participants’, say, posterior cortex. ISFC decreased with deeper sedation, much like decoding performance (Fig. 4d). Importantly, this construct is distinct from standard within-subject functional connectivity in that ISFC reflects only the statistical information associated with the task since the task is the only source of shared variance between different participants. During rest, we neither expect nor see strong ISFC (Fig. S7) since there is no common source of variance between different participants. The ISFC results can be contrasted with the within-subject functional connectivity, which remains largely unperturbed across the different levels of sedation (Fig. S7) since network structures are generally stable. Thus, ISFC seems to track sedation-dependent perceptual states.

Our results complement previous work that induced electrocortical potentials via transcranial magnetic stimulation (TMS), which differentially propagate over the neocortex as a function of the conscious state (Casali et al., 2013). Here, we leveraged passive sensory stimuli, but our findings are consistent: loss of consciousness degrades brain-wide information content via a downregulation in corticocortical information sharing. Therefore, consciousness seems a necessary condition for the presence of widespread stimulus-related cortical information. Our simple decoding approach may be sufficient to identify neural correlates of consciousness using natural sensory stimuli without needing to artificially stimulate the brain (using, e.g., TMS (Casali et al., 2013)). Neural stimulation to restore consciousness (or aspects of consciousness) may provide clues into how this information is propagated (Tasserie et al., 2022;Taylor et al., 2016).

### 3.8 Potential confounds that may account for the widespread consciousness-related information

Stimulus-related confounds, such as head motion, are likely greater when individuals are awake. However, our sedation level-dependent findings were unrelated to head motion (Fig. S8). Moreover, we observed similar results whether we used auditory cortex activity from the awake or deep anesthesia conditions (Fig. S9). This latter point is remarkable: auditory cortex activity with deep sedation is arguably “purer” than that in the awake condition, as higher-level processes and feedback loops will not modulate it, and similarly, head motion should be negligible. The generalizability of our findings across participants and levels of sedation reinforces that our results represent consciousness-related neural information rather than stimulus-related artifacts.

### 3.9 Widespread stimulus-specific information scales with consciousness in the subcortex and cerebellum

The neocortex is not the only neural structure involved in consciousness. Much has been debated regarding the role of the thalamus, other subcortical structures, and the cerebellum (e.g.,Merker, 2007;Panksepp et al., 2017;Tononi & Koch, 2015). Like the neocortex, the subcortex’s stimulus-specific information content demonstrated a dose dependence on the level of sedation. Similarly, so did the cerebellum, although its dependence on sedation level displayed a more complex relationship (Fig. S10).

## 4 Discussion

We demonstrated the existence of stimulus-specific information throughout the entire brain. By using a very simple decoder—based on*t*-statistics, the same metric commonly used in univariate analysis of brain activity—and aggregating the information contained across ~10,000 voxels, we uncovered stimulus-specific information across the entire brain, even in brain regions where univariate analysis indicates that there is approximately no stimulus-evoked signal. This analysis strictly concerned patterns of brain activity within each subject; however, we also demonstrated that the across-subject variability of brain activity can differentiate between tasks as well. Common confounds could not explain our results, which are attributable to the aggregation of information across voxels; noisier data require more aggregation. Importantly, our analyses used unbiased metrics akin to cross-validation (Efron & Tibshirani, 1997). Finally, we evaluated how this task-specific widespread information relates to conscious perception by studying the effects of sedation. In an auditory task, degradation of (1) information in brain regions outside of the primary sensory cortex and (2) intersubject correlations as a function of sedation level establishes that at least some widespread information is necessary for conscious perception. These results challenge the long-standing localizationist view that perception is linked to localized “blobs” of brain activity and has important implications regarding the current standards of fMRI analysis and interpretation.

The notion of widespread, task-specific cortical dynamics is gaining traction across multiple fields of neuroscience. A decade ago,Gonzalez-Castillo et al. (2012)used fMRI to demonstrate task-related information across the human brain, averaging 100 repetitions of the same task (3 participants, 9–10 sessions collected over 3 months) to uncover widespread information. Their rigorous work allows for voxel-level inferences but at the expense of arduous data collection efforts. In contrast, our approach reliably uncovered widespread information, even in smaller fMRI studies. Importantly, theGonzalez-Castillo et al. (2012)analysis demonstrates that transformations of the task vector can be found across the brain, while our analysis only establishes the existence of ubiquitous task-related information. Decoding studies using statistical learning approaches evidence the existence of task-specific information outside of GLM areas (Cox & Rogers, 2021;Kumar et al., 2020;Mohr et al., 2015;Rish & Cecchi, 2017;Rish et al., 2012). It has also been demonstrated that some of these local information patterns can be uncovered via multivariate statistics (Davis et al., 2014) and decoding methods (Haxby et al., 2014;Kriegeskorte et al., 2006;Norman et al., 2006), but without explicitly demonstrating the amount of information lost by ignoring subthreshold regions. Similarly, recent human and macaque monkey fMRI studies demonstrate the presence of retinotopic tuning in cortical and, in macaques, subcortical regions remote from the visual cortex (Klink et al., 2021;Liu et al., 2022), and multivariate analysis shows reinforcement signals present throughout the human cortex (Vickery et al., 2011). This fMRI evidence is complemented by wide-field calcium imaging and Neuropixels recordings in rodent models that capture mesoscopic neocortical and subcortical dynamics on a moment-by-moment basis, revealing brain-wide, task-specific activity across several cognitive domains (Gilad & Helmchen, 2020;Lab et al., 2023;Pinto et al., 2019;Ren & Komiyama, 2021;Stringer et al., 2019). Electrophysiology work in primates has also shown the ability to decode movements across several cortical neuronal populations (Carmena et al., 2003). Our results complement this prior work by demonstrating the presence of brain-wide, stimulus-specific information in human brain fMRI and the ease with which this information can be extracted from the mean and variance of brain activity.

Our findings have profound implications for task-based fMRI analysis. Null Hypothesis Significance Testing (NHST) is the dominant statistical paradigm in task fMRI studies, which involves drawing dichotomous inferences from mass-univariate GLM analyses: Is a voxel “activated” or not? Subthreshold voxels are discarded, although many of them contain task-specific information. Thus, NHST masks task-related activation to maintain type I error rates (e.g., α = 0.05) (Gonzalez-Castillo et al., 2012). Moreover, since fMRI meta-analyses typically rely on “vote-counting” procedures (Costafreda, 2009;Hedges & Olkin, 1980), regions with small, consistently subthreshold effects remain uncovered. However, these inferential issues can be at least partially overcome. On the study level, dichotomous interpretations of results, including those intrinsic to NHST, should be avoided. Analytically, taking advantage of covariance in the data and more flexible functional forms (cf. linear effects), such as the inclusion of basis or nonparametric functions, nonlinear terms, and/or temporal derivatives, may also improve sensitivity (Buchel et al., 1996,^1998^;Chen et al., 2023;Hopfinger et al., 2000). Such approaches (including the analyses presented here) are superior to standard GLM, which assumes that task-related information can be linearly projected onto the hemodynamic response function-convolved task vector. At the meta-analytic level, data sharing can facilitate mega-analyses, enabling researchers to pool raw data from many studies, and sharing unthresholded maps of the estimated effects and their standard errors can facilitate proper meta-analysis (cf. vote counting) (Costafreda, 2009). Evidently, much information is left on the table in task fMRI studies, especially in univariate analysis but also with current multivariate approaches. Our results highlight the urgent need to develop task fMRI analysis methods that can capture and interpret widespread task-related information.

At face value, the presence of brain-wide information reflects the state of consciousness rather than demonstrating its mechanisms. However, we contend that at least*some*of the brain-wide information is necessary (causal) for conscious perception. This claim is based on two premises. The first premise, which we do not evidence but believe is a reasonable postulate, is that brain activity is necessary for conscious perception. The second premise, which we do evidence, is that auditory cortex activity is insufficient for conscious perception. As a result, our findings have implications beyond neuroimaging and neuroscience in general—they extend to the study of consciousness.

Our and others’ results render the presence of task-related brain-wide information indisputable. We decoded this information using both the magnitude (ignoring individual differences) and variance (based entirely on individual differences) of brain activity. In doing so, we demonstrated the ease with which this information can be uncovered, even in brain regions that mass-univariate analyses suggest to be approximately orthogonal to the delivered stimuli. We also show that the ubiquity of this information is not without bounds—it dissipates under propofol-induced sedation. By introducing a single assumption, we imply that at least*some*of this information is necessary for consciousness. Although we only compared the brain responses to sensory stimuli, yet sensory-related information was spread across the entire brain. Neurocognitively, our results imply that perceptual states engage the entire brain. We speculate that the details of the distribution of information may define the nuanced properties of perception, for example, the affordances of the oblong, yellow object. Finally, these results strongly challenge the notion of information localization in the brain without precluding regional specialization of function. For example, although language-specific information can be uncovered across the entire neocortex (Huth et al., 2016), the crucial role of Broca’s area is incontrovertible (Broca, 1865). Unraveling the unique contribution of diverse brain regions to perceptual states requires methods beyond traditional linear, univariate analyses which will allow disclosing the necessity of some and the sufficiency of others brain regions.

In contrast to the localizationist viewpoint, it has long been argued that brain-wide information sharing must underlie conscious perception, as this sharing facilitates the coherent integration of neural information. Most notably, this idea was first stated by WilliamJames (1890)and expounded on byBaars (1988)andEdelman and Tononi (2000), and rendered into a mechanistic theory byMashour et al. (2020), which is summarized byBlock (2023)(see, however,Schurger & Graziano, 2022). Our approach and results provide a pathway to dissect different neural hypotheses of consciousness, which suggest differential regional involvement and, thus, consciousness-related information content.

## Data and Code Availability

Our custom R functions can be found on GitHub:https://github.com/avigotsky/widespread_signal. Datasets 1–4 can be found on OpenPain.org under LimitsDecoding/Data/BetaMaps. Datasets 5 and 6 can be found on OpenNeuro (ds000158 and ds003171, respectively).

## Author Contributions

A.D.V.: Conceptualization, Methodology, Software, Formal Analysis, Writing—Original Draft, Writing—Review & Editing, Visualization; R.J.: Conceptualization, Software, Writing—Review & Editing; P.B.: Conceptualization, Writing—Review & Editing; G.D.I.: Conceptualization, Investigation, Writing—Review & Editing, Supervision; M.N.B.: Investigation, Writing—Review & Editing; A.V.A.: Conceptualization, Investigation, Writing—Review & Editing, Supervision, Project administration.

## Funding

This research is partly supported by the computational resources and staff contributions provided for the Quest high-performance computing facility at Northwestern University, jointly supported by the Office of the Provost, the Office for Research, and Northwestern University Information Technology. This material is based upon work supported by the National Science Foundation Graduate Research Fellowship under Grant No. DGE-1324585. G.D.I. was supported by the ERC Consolidator Grant PAINSTRAT. This work is funded by the National Institute of Drug Abuse at National Institutes of Health (1P50DA044121) and the National Institute of Neurological Disorders And Stroke of the National Institutes of Health under Award Number F31NS126012. The content is solely the authors’ responsibility and does not necessarily represent the official views of the National Institutes of Health.

## Declaration of Competing Interest

The authors have no competing interests to declare.

## Supplementary Material

Supplementary Material
